# Characteristics of epstein barr virus variants associated with gastric carcinoma in Southern Tunisia

**DOI:** 10.1186/1743-422X-8-500

**Published:** 2011-11-03

**Authors:** Dorra BenAyed-Guerfali, Wajdi Ayadi, Imen Miladi-Abdennadher, Abdelmajid Khabir, Tahia Sellami-Boudawara, Ali Gargouri, Raja Mokdad-Gargouri

**Affiliations:** 1 Department of Molecular Genetics of Eucaryotes, Center of Biotechnology of Sfax, University of Sfax, Sidi Mansour Street, Sfax 301, Tunisia; 2 Department of Anatomo-pathology, Habib Bourguiba Hospital, El Ain Street, Sfax 3000, Tunisia

**Keywords:** Gastric carcinoma, EBV, EBER, polymorphisms

## Abstract

**Backgroud:**

EBV-associated Gastric Carcinoma (EBVaGC) has a distinct clinical features and its prevalence is variable worldwide.

**Results:**

To determine the prevalence of EBVaGC in Tunisia, EBV-encoded small RNA (EBER) expression was assessed in 81 gastric carcinoma (GC) specimens. The nuclear EBER expression was detected in 12 out of 81 GC cases (14.81%) and concordance between the score range of EBER staining and the number of EBV DNA copies as estimate by QPCR is observed. On the other hand, we found that EBVaGC strongly correlated with age at diagnosis, and weakly with tumor differentiation and venous invasion.

Furthermore, the EBVaGC specimens were subjected to determine the EBV DNA polymorphisms. Our results show a unique genetic profile of the EBV strains regarding the A and D types, the F prototype, the retention of *Xho*I restriction site and the 30 bp del-LMP1 variant. ***According to our previous studies on nasopharyngeal carcinoma (NPC), we suggested that EBV strains associated to GC and NPC shared some similarities in Tunisian patients*.**

**Conclusion:**

The prevalence of EBVaGC is of 14.81% in the southern Tunisia and that common EBV strain are associated with both NPC and GC which are likely to differ from Asian strains. Our findings support therefore a certain geographical distribution of EBV strains which is not restricted to EBV-associated malignancies.

## Background

Gastric carcinoma (GC) is the second leading cause of cancer death worldwide [1]. The incidence of GC varies from one geographic region to another, suggesting that genetic and environmental factors including *Helicobacter pylori *infection are considered to contribute to gastric carcinogenesis [2,3]. In southern Tunisia, the annual incidence of GC varies from 2.6 to 4.8/100 000 persons [4]. In the few past years, many reports have explored the association between Epstein-Barr Virus (EBV) infection and GC [5-9]. The EBV-associated GC (EBVaGC) has been evidenced by the presence of uniform expression of EBV-encoded small RNA (EBER) in GC cells [10], the detection of monoclonal EBV episomes in GC cells [11] and the increase of serum antibodies against viral capsid antigen [12]. The incidence of EBVaGC varies widely from 2 to 18% as reported by previous studies [13-20]. Moreover, the clinical features of EBVaGC include male predominance, relatively younger age and location in the proximal stomach [9,21]. The EBVaGC shows a lower rate of lymph-node involvement and has a relatively favourable prognosis compared to EBV-negative one [22].

In EBVaGC, infection of tumor cells is characterized by a type I pattern of latency, in which the expression of viral latent genes is restricted to Epstein-Barr nuclear antigen (EBNA)-1, EBER, latent membrane protein LMP-2A and transcripts from the *Bam*HI A rightward frame (BARF)-0 and -1 [11,23]. Since the EBV products of type I latent infection have been demonstrated to be involved in EBV-mediated tumorogenesis, it was suggested that the oncogenic process in EBVaGC could be driven by different mechanisms from those of conventional GC. Subsequently, EBVaGC malignancy could be considered as a different entity among GC which requires greater attention to improve patient care.

The present study was conducted with the aim to determine the prevalence of EBVaGC in southern Tunisia and subsequently to analyze specific EBV polymorphisms in the tumor isolates.

## Materials and methods

### Patient's characteristics

A total of 81 primary gastric carcinomas were collected, between January 1999 and December 2009 from patients who underwent radical surgical resection at the Department of Digestive Surgery of Habib Bourguiba University Hospital (Sfax, Tunisia). All patients gave informed consent prior to specimen collection according to institutional guidelines. None of the patients had pre-operative or post-operative chemotherapy. Clinico-pathological parameters such as gender, age, anatomical site, histological type, pathological stage, tumor size and venous invasion were evaluated by reviewing medical charts and pathological records. At the time of surgery, the age of patients ranged from 18 to 94 years (mean: 59.58 years). The anatomical site of tumor was determined according to the predominant location of the lesion as cardia (n = 8), body (n = 22), and antrum (n = 48). The histological subtypes were classified according to the criteria of Lauren [24] as intestinal type (n = 47) and diffuse type (n = 34) but also of World Health Organization as poorly differentiated (n = 43), moderately differentiated (n = 26) and well-differentiated (n = 9). The clinical stage of the disease was determined according to the tumor, node and metastasis (TNM) classification of the American Joint Committee on Cancer [25]. Our cohort contains 9 patients at stage I or II and 47 patients at stage III or IV.

### In-situ hybridization

EBV was identified by the expression of EBV-encoded small RNA (EBER). Briefly, *in situ *hybridization (ISH) assay was performed on 3 μm paraffin-block sections with EBV oligonucleotide probes complementary to the EBER-1 and -2 according to the manufacturer's instructions (***PNA ISH detection kit***, Dako Cytomation). The hybridization signals were visualized with diaminobenzidine (DAB) and positive nuclear signal was recognized as dark brown nuclear staining under light microscopy. As positive control, section from a known EBER-positive NPC tissue was used. Staining was scored from 1 to 3 according to the percentage of stained cell nuclei in tissue sections. Score 3 was attributed when positive signal was observed in more than 75%, score 2 in 75 to 50% and score 1 in less than 50% of cells.

### DNA extraction

DNA was extracted from formalin-fixed, paraffin embedded tissues. After tumor identification on hematoxylin-eosin-stained slides, tumoral areas were scraped from 40 μm thick paraffin sections. The collected materials were de-waxed by washing in xylene and rinsed in ethanol. Dried tissues were digested with proteinase K in presence of SDS at 55°C overnight, followed by phenol/chloroform extraction as described previously [26]. The quantity of DNA was checked by spectrophotometer and stored at -20°C for further use.

### PCR- RFLP and Sequencing

Five regions of the EBV genome were targeted for polymorphism analysis by PCR, RFLP or sequencing. The polymorphisms studied encompass the A or B type in the EBNA-3C gene, the C or D type in the BamHI-W1/I1 region, the prototype F or f variant in the BamHI-F region, the loss of an *Xho*I site in the first exon of BNLF1gene (*Xho*I-loss variant) and the 30 bp deletion in the third exon of the same gene (30 bp del-LMP1 variant). ***Genotyping was performed on DNA from GC patients EBV-positive and as control, we analyze 10 DNA samples from nasopharyngeal mucosa positive for EBV*.**

PCR amplification was performed on 200 ng DNA in a final reaction mixture of 50 μl containing 0.2 μM of each primer, 200 μM of dNTP, 2 mM MgCl2, 1X PCR buffer and 1 unit of Taq DNA polymerase (Fermentas). The primer sequences and the size of PCR products are shown in Table 1.

**Table 1:** Summary of primer sequences encompassing the EBV types and variants

EBV genes and regions (types or variants)	Primer sequences (5'-3')	Product size (bp)
**EBNA-3C gene** **(type A or B)**	F: AGAAGGGGAGCGTGTGTTGT R: GGCTCGTTTTTGACGTCGGC	PCR products 153: type A 246: type B

**BamH1-W region** **(type C or D)**	F: ACCTGCTACTCTTCGGAAAC R: TCTGTCACAACCTCACTGTC	PCR+ *Bam*HI digestion 205: type C 130+75: type D

**BamH1-F region** **(f variant)**	F: TCCCACCTGTTACCACATTC R: GGCAATGGGACGTCTTGTAA	PCR+ *Bam*HI digestion 198: F variant 127+71: f variant

**Exon 1 of BNLF1 gene** **(*XhoI *variant)**	F: ACAATGCCTGTCCGTGCA R: AGAAACACGCGTTACTCT	PCR+ *Xho*I digestion 497: *Xho*I- 340+157: *Xho*I+

**Exon 3 of BNLF1 gene** **(30bpdel-LMP1)**	F: TGGAGGGAGAGTCAGTCAGGC R: ATTGACGGAAGAGGTTGAAAAC	PCR products 224: 30bpdel-LMP1 254: wt-LMP1

F: Forward primer

R: Reverse primer

The C/D and F/f typing is based on the *BamHI *digestion of each PCR product. The enzymatic reactions were carried out in a final volume of 20 μl containing 10 μl of PCR product, 1X digestion buffer and 10 units of *BamHI *enzyme (Fermentas). After overnight incubation at 37°C, products were analyzed on 2% agarose gel and visualized by ethidium bromide staining, under UV illumination. The same conditions described above were assessed to identify the *XhoI *site in the first exon of BNLF1gene. DNA from B95.8 (GenBank accession No.V01555) and C666-1 cell line (GenBank accession No. ABV54173) were used as control for EBV types and variants. The C666-1 cell line derived from a Chinese NPC that harbours A/C types, f variant, *Xho*I-loss variant and 30 bp del-LMP1 variant.

DNA sequencing was performed on the purified PCR products of the third exon of BNLF1 gene using the SV gel Purification kit (Promega). Cycle sequencing was performed using the ABI PRISM Big Dye Terminator Cycle Sequencing Kit (Applied Biosystems) according to the manufacturer's instructions. All sequences were performed bi-directionally. The sequencing results were then compared with the EBV sequences of B95.8 (GenBank accession No.V01555), Chinese NPCs (Cao cells [27], C666-1 cells (GenBank accession No. ABV54173) and NPC10 biopsy [28] and Tunisian NPC specimens (CV4, CV5, and CV6 [29]).

### Real time quantitative PCR

Q-PCR assay targeting the BamHI-W region of the EBV genome was performed on the 12 EBV-positive specimens displaying nuclear staining and 6 EBV negative specimens. Reactions were carried out on an iCycler iQ™ Real-time PCR system (BioRad), using primers flanking the BamHI-W region of the EBV genome and TaqMan probe as described previously [30]. Aliquot of 1 μg DNA was used for amplification in a total reaction volume of 25 μl containing 300 nM of each primer, 25 nM of TaqMan probe, 1X PCR buffer, 2mM MgCl2, 200 μM of each dNTP and 1 unit of GoTaq Hot Start polymerase (Promega). The standard curve was prepared using serial 10-fold dilutions of the recombinant plasmid pGEMT/BamHI-W. Samples were run in duplicate and the results averaged to calculate EBV viral load expressed in copies per reaction.

### Statistical analysis

Statistical analysis was performed with SPSS 13.0 statistical software program for Windows (SPSS Inc., Chicago, Illinois, USA). P-value of less than 0.05 was considered as statistically significant.

## Results

### Prevalence of EBVaGC and association with clinico-pathological characteristics

**Figure 1:**
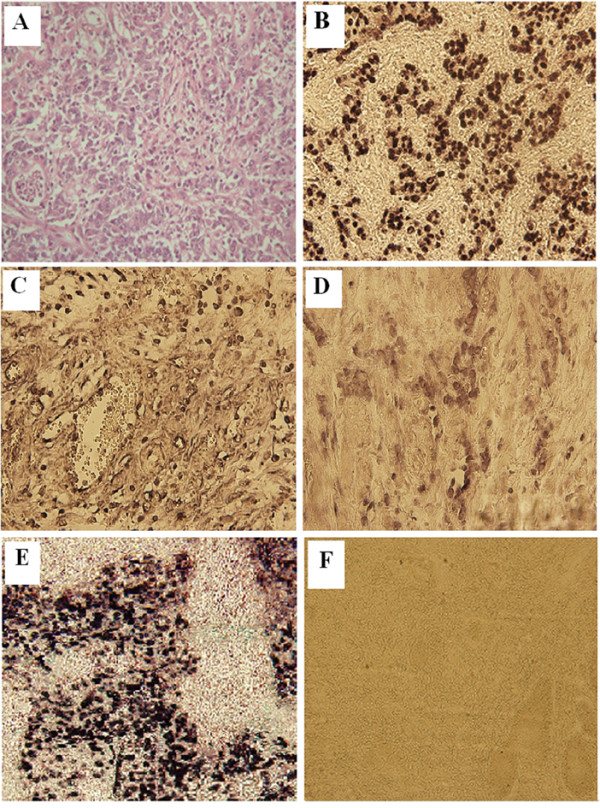
**Detection of Epstein-Barr virus encoded small RNAs (EBERs) by in-situ hybridization in gastric carcinoma tissues**. A: H&E staining. ***B: Intense EBER positive staining (immuno-score 3+) in the nuclei of tumor cells (original magnification × 40). C: Moderate EBER positive staining (immuno-score 2+) in the nuclei of tumor cells (original magnification × 40). D: Weak EBER positive staining (immuno-score 1+) in the nuclei of tumor cells (original magnification × 40)***. E: Positive control represented by a known EBER-positive NPC tissues (original magnification × 40). F: Gastric carcinoma tissues negative for EBER (original magnification × 10).

Eighty one specimens of GC were tested for EBV positivity using the EBER *in situ *hybridization. The frequency of EBVaGC was 14.8% (12 of 81) in which the EBER positive signal was restricted to the nuclei of carcinoma cells (Figure 1). ***The EBVaGC specimens have a variable percentage of stained tumor cells showing more than 75% in six cases (score 3), between 50 and 75% in three cases and less than 50% in the three remaining cases***. The EBVaGC cases were associated with age at diagnosis (*P *= 0.009, Table 2). Indeed, 9 out of the 12 patients with EBVaGC were aged between 45 to 60 years old. In addition, a trend toward association was observed with tumor differentiation and venous invasion as the statically significant value was not reached (*P *= 0.07 and *P = *0.09 respectively, Table 2).

**Table 2:** Correlation between EBVaGC and clinico-pathological parameters

Clinical Characteristics	N	EBV expression*	
		Negative (%)	Positive (%)
*Gender*			

Male	48	42(87.5)	6 (12.5)

Female	33	27(81.8)	6 (18.2)

*p*-value		0.47	

*Age*			

< 45	17	17(100)	0(0)

45-60	30	21(70)	9(30)

> 60	34	31(91.2)	3(8.8)

*p*-value		**0.009**	

*TNM*			

I-II	9	9(100)	0(0)

III-IV	47	40(87.1)	7(14.9)

*p*-value		0.216	

*Anatomical site*			

Antrum	48	41(85.4)	7(14.6)

Body	22	19(86.4)	3 (13.6)

Cardia	8	6(75)	2(25)

*p*-value		0.72	

*Differentiation *			

Poor	43	36(83.7)	7 (16.3)

Moderate	26	25(96.2)	1 (3.8)

Well	9	6(66.7)	3(33.3)

*p*-value		0.075	

*Lauren type*			

Intestinal	47	38(80.9)	9(19.1)

Diffuse	34	31(91.2)	3(8.8)

*p*-value		0.197	

*Tumor size*			

< 5 cm	9	8(88.9)	1(11.1)

> 5 cm	65	54(83.1)	11(16.9)

*p*-value		0.658	

*Venous invasion*			

Negatif	61	54(88.5)	7(11.5)

Positif	18	13(72.2)	5(27.8)

*p*-value		0.09	

* EBV expression was determined by EBER in-situ hybridization. Negative cases exhibited a score = 0 and positives cases showed a score = 1+ to 3+.

### Quantitative assessment of EBV in GC

Q-PCR assay was performed on 18 gastric carcinoma specimens; among them 12 displayed EBER positivity restricted to cells nuclei and 6 were negative.

The viral DNA copy number was estimated by the absolute quantification method using serial dilution of recombinant plasmid DNA (pGEMT/BamHI-W), as external standard. Standard curve was established by plotting the starting plasmid copy number against the threshold cycle (Ct), showing a linear quantification over a range from 10^6 ^to 10 copies per reaction.

**Figure 2:**
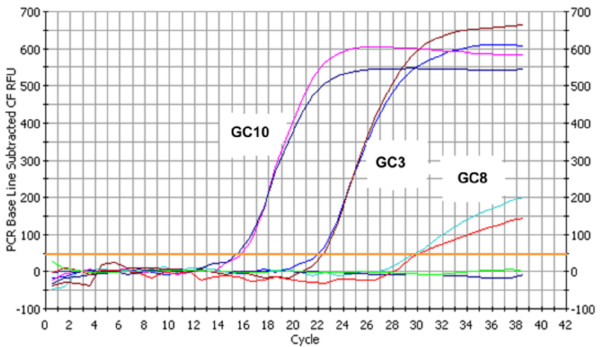
**Determination of EBV DNA copy number by Q-PCR**. Representative examples of GC cases with high (GC10), intermediate (GC3) and low (GC8) EBV DNA copies number. The horizontal line marks the threshold used for assessment of C_t _value.

Representative examples are shown on Figure 2 where cases GC10 and GC3 (Ct = 15 and 22 respectively) display strong EBER nuclear staining whereas GC8 (Ct = 30) exhibits a weak EBER expression (Figure 2). The Q-PCR data is in concordance with EBER *in situ *hybridization result. Indeed, the 6 specimens with intense EBER staining (score 3+), showed high EBV DNA copy number whereas cases with weak EBER expression (score 1+) corresponded to low viral DNA copy (Table 3).

**Table 3:** EBV DNA copy number and EBER staining score in the 12 EBV positive specimens and 6 negative cases

GC Cases	Histological Type	Tumor Site	Ct* (average)	EBV DNA * Copies/μg	EBER staining score
**10**	Intestinal	Antrum	15	6.10^5^	3+

**3**	Intestinal	Antrum	22	4.10^3^	3+

**12**	Intestinal	Antrum	23	10^3^	3+

**9**	Diffuse	Antrum	22.8	10^3^	3+

**2**	Intestinal	Body	25	5.10^2^	3+

**4**	Intestinal	Antrum	26	10^2^	3+

**6**	Intestinal	Proximal	27	8.10^1^	2+

**7**	Diffuse	Body	28	5.10^1^	2+

**11**	Intestinal	Proximal	28	5.10^1^	2+

**1**	Intestinal	Antrum	30	< 10	1+

**5**	Intestinal	Antrum	30	< 10	1+

**8**	Diffuse	Body	30	< 10	1+

**13**	Diffuse	Antrum	ND	ND	0

**14**	Diffuse	Body	ND	ND	0

**15**	Diffuse	Antrum	ND	ND	0

**16**	Diffuse	Antrum	ND	ND	0

**17**	Intestinal	Antrum	ND	ND	0

**18**	Intestinal	Body	ND	ND	0

* EBV DNA copy number was estimated by Q-PCR using the absolute quantification method. Ct: threshold Cycle. ND: Non-Determined

### Genotyping of EBVaGC strains

**Figure 3:**
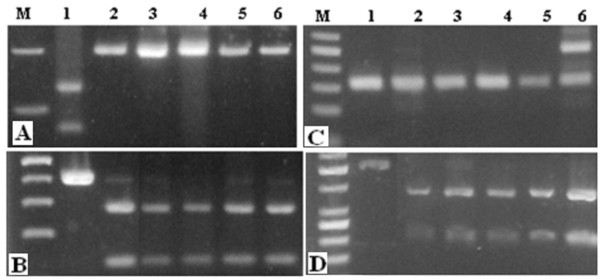
**Genotyping of EBVaGC strains**. **A: **PCR -RFLP of the BamHI-F region showing the f variant (presence of two bands of 128 bp and 71 bp) for the control C666-1 cell line (lane 1) and one band of 199 bp for the F variant for cases GC2 to GC6 (lane 2 to 6). **B: **PCR-RFLP of the BamHI-W1/I1 region. DNA fragments were digested with *BamHI *giving two bands of 139 and 67 bp (type D) for GC2 to GC6 (lane 2 to 6) or one band of 245 bp (type C) for the control C666-1 cell line (lane 1). **C: **EBNA3C region. EBV strains of types A and B correspond to DNA fragment of 153 and 246 bp respectively. Case GC6 shows dual infection with both types A and B viruses. B95.8 is a type A virus that was used as a control (lane 1). **D: ***XhoI *polymorphism in exon 1 of the BNLF1 gene. PCR product was digested by *XhoI *to yield two bands of 343 bp and 154 bp in *XhoI*+ variant for GC2 to GC6 (lane 2 to 6). C666-1 cell line is a positive of loss-*XhoI *variant (lane 1).

To further investigate the EBVaGC in our 12 Tunisian specimens, we have examined the genetic profile of EBV strains determining different types and variants. A unique profile in all studied specimens is observed (Figure 3). It was characterized by the A/D types, prototype F, retention of *Xho*I restriction site and 30 bp del-LMP1 variant. We note that the A and B types shaping two different EBV strains were found in one case of EBVaGC specimens (Figure 3).

**Figure 4:**
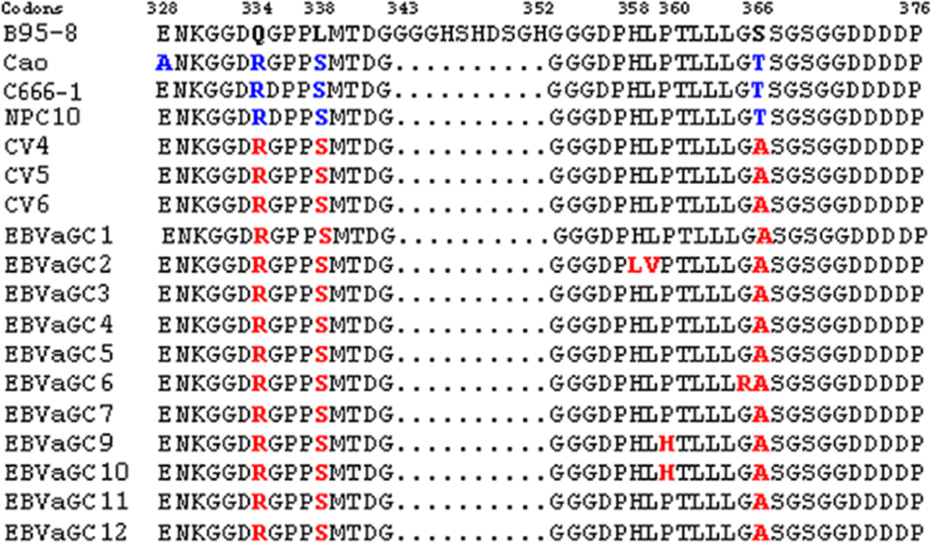
**Comparison of deduced amino acid sequences from Tunisian GC with B95.8 prototype and previously published LMP1 sequences: Cao: NPC cell line from Shangai **[27]**; C666-1 (GenBank accession No**. ABV54173) **NPC 10 from Hong Kong **[28]**and Tunisian NPC specimens: CV4, CV5, CV6 **[29]. Symbols (---) indicate amino acid deletion.

The EBV DNA analysis was also performed by partial sequencing on the third exon of the BNLF1 gene encoding amino acids 328 to 376 of the LMP1 protein. In order to confirm the 30 bp deletion encoding amino acids 343 to 352 of the LMP1 protein, sequence alignment revealed seven amino-acids changes compared to the reference strain B95.8. It was constantly found at codons Q334R, L338S and S366A in all EBVaGC specimens. The remaining amino acids substitutions, however, were especially found in one or two EBVaGC specimens: H358L and L359V (GC2), P360H (GC9-10) and G365R (GC6). Regarding our previous data on Tunisian NPC specimens, we found similar results about the three constant amino-acids substitutions. In addition, the EBV strains harbouring S366A substitution appear more associated to Tunisian specimens when compared to those defined in China (Figure 4).

*In healthy EBV carriers, polymorphism analysis showed also the A and D types, prototype F and XhoI+ variant, as similar to identify in EBVaGC specimens (data not shown)*.

## Discussion

In the present study, 81 cases of GC from patients of the south region of Tunisia were investigated for the presence of EBV and the prevalence of EBVaGC was 14.8% (12 out of 81). It was well known that the prevalence of EBVaGC varies widely from one geographical region to another and the highest frequency was noted in Germany (18%) whereas the lowest one (3.9%) was detected in Peru [13-20,31].

The EBER *in situ *hybridization was the most reliable method reported in the literature to detect latent EBV in GC. In fact, all the above mentioned studies were conducted following this method, including our study. ***The variable proportions of nuclear tumor cells that express EBER showing in our study were also deduced by Truong et al., who suggest to be related with EBV infection occurs in oncogenic process of EBVaGC ***[32]. ***However, further investigations must be conducted to clarify this observation*.**

In addition, we showed that the heterogeneous data of EBER expression could be correlated with the number of EBV DNA copies as estimated by Q-PCR supporting that this method is reliable as reported previously [33].

With regards to the clinico-pathological features of EBVaGC, strong association with age at diagnosis was observed (*P *= 0.009, Table 3). In fact, the EBVaGC was more frequently found in the group of patients aged between 45 and 60 years old, which is in line with previous studies [19,34]. No other statistical association was found, except a tendency with tumor differentiation and venous invasion (*P *= 0.075 and *P *= 0.09 respectively). ***Previous reports have indicated that EBVaGC were frequent in male, proximal stomach and tumors of diffuse type ***[9,14,16,35-39].***Recently, in a large meta-analyse, Carmago et al., confirms these associations in addition to the age at diagnosis ***[38]. ***In our study, we didn't find difference in the distribution of EBV in male compared to female patients, in proximal vs distal stomach and in diffuse vs intestinal histotype. These variations between data could be explained by the contribution of local risk factors in the pathogenesis of EBV and also by the size and charasteristics of the cohort***.

Polymorphism analysis of the 12 EBVaGC cases show exclusively the type D, prototype F, *Xho*I-retention and the 30 bp del-LMP1 variant. Concerning the polymorphism of the EBNA-3C gene, we found the A type in all EBVaGC cases and a combination of types A and B was found in only one case. These findings are in agreement with recent study ***conducted ***on four EBVaGC cases from the ***central ***region of Tunisia [34].

The predominance of type A and prototype F was also shown in previous reports independently to the geographic origin of EBVaGC as in southern China [35], southern Japan [40], and Latin American countries [41]. Interestingly, the variant-f was especially described in NPC associated EBV strains of southern Asia [42]. In this area, a predominance of C type and loss-*XhoI *variant was observed in patients with EBVaGC or NPC [35,40], in contrast to our present finding and previous data on Tunisian NPC patients [29,43].

***To better define the genotype of EBVaGC strains and compare them with those associated with the NPC, we carried out the partial sequencing of exon 3 of the BNLF1 gene and compared them ***to the prototype B95-8 and others published sequences from Asian and Tunisian NPC EBV strains [29,43]. The Q334R, L338S and L338P were found in all our EBV isolates and were already reported in EBV strains associated to NPC originating from China or Tunisia [29,44]. However, the S366A is specific to EBV isolates associated with Tunisian NPC and GC.. ***Indeed, and based on the previous report of Edwards et al.*, **[44]***describing seven phylogenetically distinct strains of LMP1, we can propose that Tunisian isolates constitute a supplementary group with a specific signature (T366A) but this hypothesis needs confirmation by sequencing of the entire BNLF1 gene in these isolates. According to this date, we suggest that polymorphism in the BNLF1 gene constitute an additional argument in line with the others EBV polymorphisms (D type, prototype F and XhoI+) supporting the dissimilarity of EBV strains between the two geographic regions*.**

On the other hand, we have described *previously *others del-LMP1 variants (69 bp and 81 bp deletion spanning codons 334-353 and 345-371, respectively) in Tunisian NPC patients [29,43]. These variants were not found and only the 30 bp del-LMP1 variant was identified in all EBVaGC cases as already reported by Chen et al., in a large study conducted on Chinese GC patients [35].

***Our polymorphism analysis of EBV isolates in healthy carriers revealed an identical genotype to those of EBVaGC and NPC suggesting that common strains are geographically distributed but not associated with a specific malignancy. In fact, the development of EBV associated malignancies could be correlated to variation in potential immune recognition in distinct populations and individuals, as proposed by Edwards et al*, **[44].

## Conclusion

The prevalence of EBVaGC in patients from southern Tunisia is 14.8% which is in range with reported data. The EBVaGC was predominantly found in the group of patients aged from 45 to 60 years old and that EBV DNA level reflect the EBER status in gastric carcinoma.

Furthermore, EBV strains associated to Tunisian patients with GC or NPC share some similarities suggesting that probably the same EBV strain are associated with both tumors. Altogether, our findings support the different geographical distribution of EBV strains, but not their restriction to an associated malignancy.

## Competing interests

The authors declare that they have no competing interests.

## Authors' contributions

DBG carried out the molecular genetic studies, EBER in situ-hybridization and drafted the manuscript. WA carried out the Q-PCR, and drafted the manuscript. IMA contributed to sequence alignment, and drafted the manuscript. AK participates to the EBER in situ- hybridization and carried out the pathological workup. TSB participate to the design of the study. AG participates to the design of the study and drafted the manuscript. RMG carried out the statistical analysis, conceived the study and wrote the paper. All authors read and approved the final manuscript.
